# Chronic psychological stress promotes breast cancer pre-metastatic niche formation by mobilizing splenic MDSCs via TAM/CXCL1 signaling

**DOI:** 10.1186/s13046-023-02696-z

**Published:** 2023-05-20

**Authors:** Yifeng Zheng, Neng Wang, Shengqi Wang, Juping Zhang, Bowen Yang, Zhiyu Wang

**Affiliations:** 1 State Key Laboratory of Dampness Syndrome of Chinese Medicine, The Second Affiliated Hospital of Guangzhou University of Chinese Medicine, Guangzhou, 510006 China; 2 Integrative Research Laboratory of Breast Cancer, Discipline of Integrated Chinese and Western Medicine, The Second Clinical College of Guangzhou University of Chinese Medicine, Guangzhou, Guangdong 510006 China; 3 Guangdong-Hong Kong-Macau Joint Lab on Chinese Medicine and Immune Disease Research, Guangzhou University of Chinese Medicine, Guangzhou, Guangdong 510006 China; 4 Guangdong Provincial Key Laboratory of Clinical Research on Traditional Chinese Medicine Syndrome, Guangdong Provincial Academy of Chinese Medical Sciences, Guangdong Provincial Hospital of Chinese Medicine, Guangzhou, Guangdong 510006 China; 5 The Research Center for Integrative Medicine, School of Basic Medical Sciences, Guangzhou University of Chinese Medicine, Guangzhou, Guangdong 510006 China

**Keywords:** Chronic psychological stress, Breast cancer, Pre-metastatic niche, Myeloid-derived suppressor cells, CXCL1/CXCR2

## Abstract

**Background:**

Emerging studies have identified chronic psychological stress as an independent risk factor influencing breast cancer growth and metastasis. However, the effects of chronic psychological stress on pre-metastatic niche (PMN) formation and the underlying immunological mechanisms remain largely unknown.

**Methods:**

The effects and molecular mechanisms of chronic unpredictable mild stress (CUMS) on modulating tumor-associated macrophages (TAMs) and PMN formation were clarified by multiplex immunofluorescence technique, cytokine array, chromatin immunoprecipitation, the dual-luciferase reporter assay, and breast cancer xenografts. Transwell and CD8^+^ T cytotoxicity detection were used to analyze the mobilization and function of myeloid-derived suppressor cells (MDSCs). mCherry-labeled tracing strategy and bone marrow transplantation were applied to explore the crucial role of splenic CXCR2^+/+^ MDSCs facilitating PMN formation under CUMS.

**Results:**

CUMS significantly promoted breast cancer growth and metastasis, accompanied by TAMs accumulation in the microenvironment. CXCL1 was identified as a crucial chemokine in TAMs facilitating PMN formation in a glucocorticoid receptor (GR)-dependent manner. Interestingly, the spleen index was significantly reduced under CUMS, and splenic MDSCs were validated as a key factor mediating CXCL1-induced PMN formation. The molecular mechanism study revealed that TAM-derived CXCL1 enhanced the proliferation, migration, and anti-CD8^+^ T cell functions of MDSCs via CXCR2. Moreover, CXCR2 knockout and CXCR2^−/−^MDSCs transplantation significantly impaired CUMS-mediated MDSC elevation, PMN formation, and breast cancer metastasis.

**Conclusion:**

Our findings shed new light on the association between chronic psychological stress and splenic MDSC mobilization, and suggest that stress-related glucocorticoid elevation can enhance TAM/CXCL1 signaling and subsequently recruit splenic MDSCs to promote PMN formation via CXCR2.

**Graphical Abstract:**

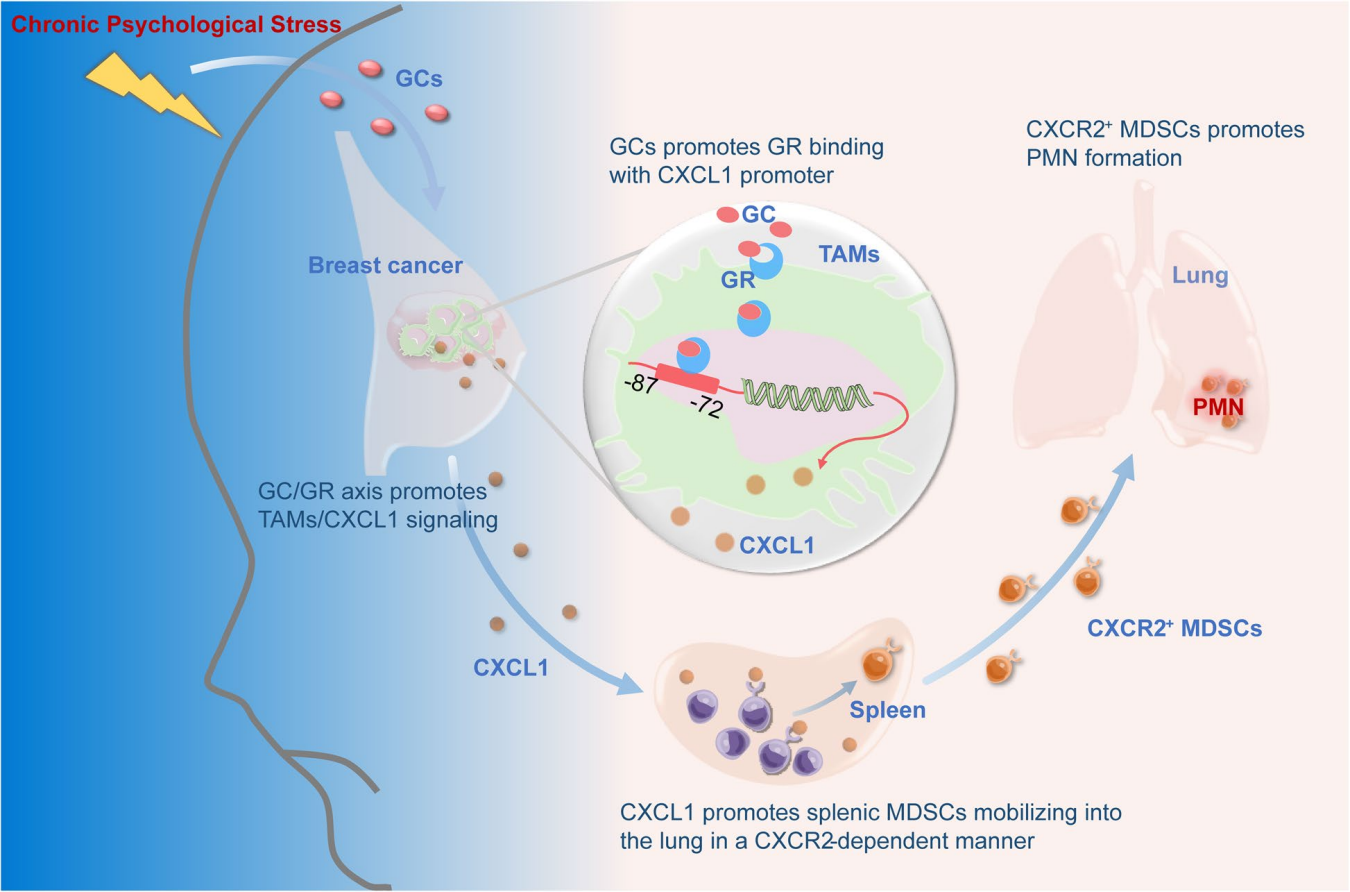

## Background

Breast cancer remains a significant public concern, with an estimated 287,850 new cases and 43,250 deaths among women annually [1]. Metastasis accounts for approximately 90% of breast cancer deaths, and 10% to 50% in situ breast carcinomas develop into invasive phenotypes [2, 3]. Therefore, it is necessary to urgently explore the key factors influencing the progression and mortality of breast cancer. Emerging studies have indicated that chronic stress-related psychological disturbances, such as depression, anxiety, and post-traumatic stress disorder, are strongly correlated with breast cancer risk [4]. An epidemiological analysis revealed that women suffering traumatic life events have a twofold higher tendency to develop breast cancer [5, 6]. Depression and anxiety, either alone or in combination, was found to be an independent indicator of recurrence and mortality in a systematic analysis involving 282,203 breast cancer patients [7]. Therefore, it is necessary to urgently explore the underlying molecular mechanisms between chronic psychological stress and breast cancer progression.

Chronic psychological stress has been shown to induce tumor immune evasion, making individuals more susceptible to malignancies and resistant to existing anti-breast-cancer therapies [8]. In a pilot study involving 116 patients with invasive breast cancer, Andersen et al*.* reported that high levels of psychological distress were related to the decreased proliferation and function of NK cells as well as T lymphocytes [9]. Additionally, Mundy-Bosse et al*.* pointed out that chronic stress would promote the generation and accumulation of myeloid-derived suppressor cells (MDSCs), while acute stress might exert the opposite effect on MDSC numbers in breast cancer patients [10]. These findings are consistent with those of preclinical studies showing that chronic stress-induced immunosuppression facilitated cancer progression by upregulating MDSCs, regulatory T cells, and M2 tumor-associated macrophages (TAMs), downregulating effector T cells and natural killer cells [11]. In addition, note that chronic stress profoundly influences tumor development through the persistent release of stress hormones and neurotransmitters. Obradović et al*.* demonstrated that stress-related hormone glucocorticoids (GCs) could promote breast cancer invasiveness by interacting with the glucocorticoid receptor (GR) [12], further suggesting the underlying correlations between psychological disorder and breast cancer progression. Several studies also reported that continuous GCs treatment was associated with decreased CD4^+^ and CD8^+^ T cells, B cells, and impaired dendritic cell function in cancer patients [13, 14]. Moreover, GR was demonstrated as a significant target of poor overall survival and relapse-free survival in estrogen receptor-negative (ER) breast cancer patients [15, 16]. Notably, single-cell sequencing data revealed higher GR expression on immune cells compared to cancer and stromal cells [17]. It is interesting to elucidate the potential effects of GC/GR axis on the tumor immune microenvironment, particularly for pre-metastatic niche (PMN) formation.

PMN has been regarded as an alternative paradigm for promoting metastasis, and recognized as a microenvironment in distant organs that sustain the survival and growth of primary cancer cells before their settlement in target sites. The formation of PMN is determined by two critical factors: bone-marrow-derived myeloid cells (BMDCs) and primary tumor-derived components [18]. MDSCs make up the majority of BMDCs within PMN, which belong to a heterogeneous subpopulation of immature myeloid cells with suppressive properties on immune cells such as T cells, dendritic cells, and natural killer cells [19]. Primary tumor-derived components include tumor-derived secreted factors (TDSFs), extracellular vesicles, and other molecular components. TDSFs presenting as a variety of pro-inflammatory cytokines, chemokines, and angiogenic factors are thought to act mainly by recruiting and colonizing MDSCs into distant tissues for initiating immune evasion and cancer metastasis [20, 21]. C–X–C motif ligand 1 (CXCL1) is one kind of TDSF. An increase in CXCL1 levels in breast cancer correlates with larger tumors, higher-grade malignancies, and shorter survival times for breast cancer patients [22]. In murine breast cancer xenografts, neutrophil infiltration within the tumor microenvironment was recruited by CXCL1 secreted by mesenchymal stromal cells (MSCs), subsequently resulting in metastasis [23]. Wang et al*.* revealed that colorectal-cancer-secreted vascular endothelial growth factor A (VEGF-A) could stimulate TAMs to produce CXCL1, which led to the accumulation of MDSCs in pre-metastatic liver tissue and subsequent metastasis [24]. In a study by the same group, CXC chemokine receptor 2 (CXCR2) was required for homing granulocytic MDSCs from the circulatory system to colitis-associated tumors [25]. Additionally, Yang et al*.* demonstrated that targeted depletion of CXCR2 in myeloid cells improved anti-tumor immunity by reducing the infiltration of MDSCs and increasing the cytotoxic activities of CD8^+^ T cells [26]. Given that PMN components might provide insight into the early prognosis of invasive breast cancer, it is of significance to investigate the underlying mechanisms regulating the CXCL1-induced accumulation of MDSCs.

A growing body of experimental evidence shows that MDSCs are primarily responsible for PMN formation, although neutrophils, macrophages, and Tregs are also involved [19]. Beyond tumor-derived components, MDSCs act primarily on peripheral lymphoid organs, such as the spleen [27]. Although MDSCs share similar phenotypes and morphologies, their quantitative and functional variety is largely dependent on cancer types, tumor stage/progression, and the anatomical location. Generally, MDSCs positively express cell surface markers CD33 and CD11b and have differential expressions of monocytic and granulocytic markers (CD14 and CD15, respectively) in humans. In tumor-bearing mice, MDSCs are distinguished by surface markers CD11b and GR-1, and GR-1 includes the isoforms Ly6C and Ly6G [28]. CD11b^+^/GR1^+^ MDSCs represent < 3% of all nucleated splenocytes in tumor-free mice, while MDSCs within the spleen expand dramatically to over 20% in tumor-bearing mice [29]. This phenomenon agrees with that observed by Youn et al*.*, who extended the analysis of the crucial role of spleen-derived MDSCs in 10 different cancer models. It was found that a majority of the MDSCs belonged to the granulocytic CD11b^+^Ly6C^lo^Ly6G^+^ (G-MDSC) phenotype [30]. Notably, although sharing the common markers CD11b, Ly6G, and Ly6C, splenic MDSCs exhibited distinctive functions compared to their tumor-derived counterparts, producing higher amounts of reactive oxygen species and suppressing antigen-specific T cells [31]. Hypoxia induction would precipitate splenic MDSCs to form non-specific suppressor cells and differentiate into macrophages highly secreting IL-10, ARG1, iNOS, IL-12, and IL-6 [27, 29]. In the 4T1-bearing mice model, a substantial increase of splenic CD11b^+^/GR1^low^ and CD11b^+^/GR1^high^ cells was observed throughout breast cancer progression [32]. Moreover, 4T1-bearing mice treated with docetaxel showed a decrease in splenic MDSCs and polarization to anti-tumorigenic M1-like macrophages [33]. However, the mechanisms associated with splenic MDSC recruitment in the PMN remain unclear.

Here, we investigated the influence of chronic psychological stress on PMN formation in breast cancer xenografts. We found that chronic psychological stress could enhance TAM/CXCL1 signaling in the breast cancer microenvironment via the GC/GR axis. Further, CXCL1 could subsequently mobilize splenic MDSCs into the lung to establish PMN in a CXCR2-dependent manner. These results shed new light on the role of spleen immunity in mediating chronic psychological stress-mediated cancer progression and provide CXCL1-CXCR2 signaling as a potential drug target for preventing PMN formation.

## Materials and methods

### Cell culture

4T1 murine breast cancer cell line (RRID: CVCL_0125) and RAW264.7 cell line (RRID:CVCL_0493) were purchased from KeyGEN BioTECH (Nanjing, China) and maintained in Dulbecco’s Modified Eagle Medium (DMEM, Gibco, Grand Island, NY, USA) and RPMI 1640 medium (Gibco, Grand Island, NY, USA), respectively. Both cell lines were supplemented with 10% fetal bovine serum (Gibco, Grand Island, NY, USA) and 1% penicillin and streptomycin (Gibco, Grand Island, NY, USA) and incubated at 37**º**C in a humidified incubator containing 5% CO_2_. 20 ng/mL IL-4 (PeproTech, NJ, USA) and 20 ng/mL IL-10 (PeproTech, Cranbury, NJ, USA) were added into the culture medium of RAW264.7 to obtain M2-like macrophages. All cell lines were authenticated using short tandem repeat profiling and tested for mycoplasma contamination.

### Flow cytometry sorting

CD8^+^ T cells were sorted from the spleen of BALB/c mice. MDSCs were sorted from the spleen of 4T1 tumor-bearing mice, and M2-TAMs were sorted from the tumor. Single-cell suspensions were prepared and stained with the appropriate antibodies. Cell sorting was performed on a FACS Aria III flow cytometer (BD Biosciences, San Jose, CA, USA). CD8^+^ T cells were labeled with CD8-APC/Cyanine7 (BioLegend Cat# 100,714, RRID:AB_312753), and MDSCs were marked with Gr-1-Alexa Fluor 488 (Thermo Fisher Scientific Cat# 53–5931-80, RRID:AB_469917), and CD11b-PE (BioLegend Cat# 101,208, RRID:AB_312791). M2-TAMs were stained with F4/80-APC (Thermo Fisher Scientific Cat# 17–4801-82, RRID:AB_2784648) and CD206-PE-Cyanine7 (Thermo Fisher Scientific Cat# 25–2069-42, RRID:AB_2573426).

### 5-ethynyl-2’-deoxyuridine (EdU) assay

The sorted MDSCs were assayed for proliferation using the Cell-Light Apollo 567 Staining Kit (RiboBio, Guangzhou, China) according to the manufacturer’s instructions. Briefly, MDSCs were labeled with the EdU reagent and then prepared for smears. The smears were air-dried and fixed in 4% paraformaldehyde. The cell nuclei were stained with Hoechst33342 after 30 min incubation with Apollo staining reaction solution and pictured by fluorescence microscopy (Nikon Eclipse Ts2R, Nikon, Japan). Triplicate independent experiments were repeated.

### Carboxyfluorescein-succinimidyl-ester (CFSE) dilution assay

The proliferation of CD8^+^T cells was determined using the CellTrace CFSE cell proliferation kit (Thermo Fisher Scientific, Hudson, NH, USA) according to the manufacturer’s instructions. Briefly, CD8^+^T cells were resuspended in phosphate buffer saline (1 × 10^6^/mL) and then incubated with CFSE reagent for 20 min at 37**º**C. The staining was terminated by adding a complete culture medium. Flow cytometry analysis was performed after 72 h incubation. Triplicate independent experiments were repeated.

### Western blotting

For sodium dodecyl sulfate polyacrylamide gel electrophoresis (SDS-PAGE), to adapt with molecular weight variations among target proteins, different percentage of polyacrylamide gel was prepared. Particularly, a 15% gel was used for CXCL1 detection, while a 10% gel was employed for both the glucocorticoid receptor and β-actin. Following SDS-PAGE separation, the proteins were transferred to polyvinylidene fluoride (PVDF) membranes (Millipore, Billerica, MA, USA) and probed with the primary antibodies, including glucocorticoid receptor antibody (Proteintech Cat# 24,050–1-AP, RRID:AB_2813890), CXCL1 (Proteintech Cat# 12,335–1-AP, RRID:AB_2087568), β-actin antibody (Cell Signaling Technology Cat# 4970, RRID:AB_2223172). Following secondary antibodies incubation, an advanced ECL luminescence reagent (Tanon Science & Technology, Shanghai, China) was used, and optical density measurement was taken by ImageLab software (BIO-RAD, Hercules, CA, USA). Triplicate independent experiments were repeated.

### Plasmid transfection

The shRNA plasmids targeting *CXCL1* and *GR* were purchased from Vigene Biosciences (Jinan, China). As directed by the manufacturer, all plasmids were transfected using lipofectamine 3000 (Invitrogen, Carlsbad, CA, USA).

### Transwell invasion assay

The transwell assay was performed using 24 well chambers coated with matrigel (356234, Corning, NY, USA). The cells were quantified and seeded into the upper compartment. Cells that penetrated the filter were fixed with 4% paraformaldehyde and stained for 20 min with 0.1% Coomassie blue. Triplicate independent experiments were repeated.

### Cytokine array assay

The mouse cytokine array (RayBio, Norcross, GA, USA) was used to detect cytokines secreted by M2-type TAMs. Briefly, the cytokine antibody-coated membranes were blocked with a blocking buffer for 30 min and incubated with a culture medium of the sorted M2-type TAMs overnight at 4 °C. Then, the membranes were washed and incubated with the biotin-conjugated detection antibody cocktail and the horseradish peroxidase (HRP)-conjugated streptavidin. Finally, the membranes were washed, subjected to chemiluminescence, developed, and photographed.

### Quantitative real-time PCR

Total RNA was extracted with an RNAiso Plus Reagent (Takara BIO, Shiga, Japan). cDNA was prepared using PrimeScript RT reagent Kit with gDNA Eraser (Takara BIO, Shiga, Japan). Quantitative PCR was performed using SYBR Premix Ex TaqII (Takara BIO, Shiga, Japan) by the Biosystems QuantStudio 7 Flex Real-Time PCR System (Thermo Fisher Scientific, Hudson, NH, USA). The amplifications were performed in triplicate and normalized to β-actin. The 2-^ΔΔCt^ method was used to calculate relative gene expression levels. Primer sequences are as follows: CXCL1 forward primer CTGGGATTCACCTCAAGAACATC, CXCL1 reverse primer CAGGGTCAAGG-CAAGCCTC; β-actin forward primer GGCTGTATTCCCCTCCATCG, β-actin reverse primer CCAGTTGGTAACAATGCCATGT. Triplicate independent experiments were repeated.

### Luciferase reporter assays

The CXCL1 promoter fragments were amplified and cloned into a pGL3-Basic vector carrying the Gaussia luciferase (GLuc) and secreted alkaline phosphatase (SEAP) reporter gene (GeneCopoeia, Rockville, MD, USA). M2-type RAW264.7 cells were transfected with the CXCL1 promoter-luciferase reporter plasmids. Following transfection, Secrete-Pair Dual Luminescence Assay Kit (LF001, GeneCopoeia, Rockville, MD, USA) was used to measure luciferase activity in the supernatant. Luciferase activity was normalized using SEAP activity. Triplicate independent experiments were repeated.

### Chromatin immunoprecipitation (ChIP) assay

ChIP assays were performed using the ChIP assay kit (P2078, Beyotime Biotechnology, Shanghai, China) according to the manufacturer’s instructions. Cells were washed with ice-cold PBS and crosslinked with 1% formaldehyde for 30 min at 37 °C, followed by quenching with glycine. The sonicated cell lysates were immunoprecipitated with glucocorticoid receptor antibody (Proteintech Cat# 24,050–1-AP, RRID:AB_2813890) and IgG (Abcam Cat# ab172730, RRID:AB_2687931). Primers were designed based on the predicted GR binding sites on the CXCL1 promoter using hTFtarget database (Forward primer AGACTCTGAAGTCTCACTACTCC; Reverse primer GCTGGAACTGGTTAGAGGCT). Finally, the DNA obtained by immunoprecipitation was subjected to PCR. Triplicate independent experiments were repeated.

### Mice procedures and ethics

Female Balb/c mice at five weeks of age were obtained from the Beijing Vital River Laboratory Animal Technology (Beijing, China). CXCR2-deficient (CXCR2^KO^) mice on a Balb/c genetic background were constructed by Gempharmatech (Nanjing, China). All mice were treated according to experimental protocols approved by the Institutional Animal Care and Use Committee at Guangdong Provincial Hospital of Chinese Medicine (ethics approval number: 2019061). The orthotopic mouse model of breast cancer under CUMS was performed according to our previous study [34]. Tumor volume was measured every three days and calculated with the formula ([width] ^2^ × [length]/2). According to international animal welfare recommendations, tumors that reached 20 mm in diameter were used as the experimental endpoint, at which mice were humanely euthanized. Behavioral changes were examined after 4 weeks of CUMS, including open field and sucrose preference tests. The bioluminescence of the lung colonization was imaged and quantified with the IVIS-Spectrum system (PerkinElmer, Boston, MA, USA). To observe the role of TAMs in breast cancer progression under CUMS, clodronate liposomes (Yeasen Biotech, Shanghai, China) were used to delete macrophages in vivo by intraperitoneal injection of 150 μl, followed by administration of 100 ul every two weeks. Splenectomy was performed in mice to assess the role of the spleen in CUMS-induced PMN formation. In addition, spleen-derived MDSCs from CXCR2^KO^ or CXCR2^WT^ tumor-bearing mice were sorted and cultured in 6-well plates. 50 μl lentiviral vectors expressing mCherry (GeneCopoeia, Rockville, MD, USA) and 5 μg/ml polybrene reagent (GeneCopoeia, Rockville, MD, USA) were added into the culture medium. After 15 h of co-culture, MDSCs were labeled with mCherry. Then, mCherry-MDSCs were injected into the spleen of the recipient mice to trace the recruitment of exogenous spleen-derived MDSCs.

### Multiplex immunofluorescence staining

Paraffin-embedded sections of tumor tissues from mice were dewaxed according to the conventional immunohistochemical methods. The sections were then performed multiplex immunofluorescence staining using Opal 7-color Manual IHC Detection Kit (NEL811001KT, Akoya Biosciences, Marlborough, MA, USA) according to the manufacturer’s instructions. Briefly, after tissue blocking with 10% goat serum, primary and the HRP-conjugated secondary antibodies were applied sequentially, and then the tyramine signal amplification (TSA) was performed. The process was repeated until all primary antibodies were applied. Primary antibodies included Pan-Keratin antibody (Cell Signaling Technology Cat# 4545, RRID:AB_490860), CD11b antibody (Abcam Cat# ab133357, RRID:AB_2650514), CD8 antibody (Abcam Cat# ab217344, RRID:AB_2890649), FoxP3 antibody (Cell Signaling Technology Cat# 12,653, RRID:AB_2797979), CD206 antibody (Cell Signaling Technology Cat# 24,595, RRID:AB_2892682), Ly6g antibody (Abcam Cat# ab238132, RRID:AB_2923218). Each antibody was visualized by treatment with Opal 520 TSA, Opal 540 TSA, Opal 570 TSA, Opal 620 TSA, Opal 650 TSA, and Opal 690 TSA. After each TSA operation, the slides were subjected to microwave heat treatment to remove the antibodies. The above procedure was repeated for each primary antibody staining. The nuclei were stained with DAPI after all the antigens were labeled. The slides were scanned using the Vectra Polaris Automated Quantitative Pathology Imaging System (Akoya Biosciences, Marlborough, MA, USA) to obtain multispectral images. Quantification was performed using inForm software (Akoya Biosciences, Marlborough, MA, USA).

### Establishment of bone marrow chimera

Bone marrow transplantation was used to create chimeric mice. Briefly, recipient mice were sub-lethally irradiated (8 Gy) using MultiRad 225 X-ray irradiation system (Faxitron, Lincolnshire, IL, USA). Bone marrow cells from donor mice were resuspended at a concentration of 1 × 10^8^/mL. A 100 μl aliquot was injected into mice that had been irradiated. From one day before irradiation until two weeks after, mice were given antibiotic water that contained trimethoprim-sulfamethoxazole.

### Mice behavioral analysis

Individual mice were placed in an arena of 40 × 40 × 40 cm for the open field test. A camera continuously recorded the activity traces of the mouse for 6 min. The total movement distance and immobility time were analyzed by the Smart 3.0 software (Panlab, Cornella, Spain). For the sucrose preference test [35, 36], mice were adapted to 1% sucrose solution for 2 days, they were exposed to one bottle of water and one bottle of 1% sucrose solution, with the exchange of bottle position every 12 h. The mice were subsequently deprived of water and food for 24 h, followed by the formal test. The consumption of water and 1% sucrose within 4 h was calculated. Sucrose preference (%) = sucrose consumption (mL) / [sucrose consumption (mL) + water consumption (mL)] × 100%.

### Hematoxylin–eosin staining

The tissue sections were deparaffinized and hydrated. The cell nucleus was visualized with 10% hematoxylin and the cytoplasm with 1% eosin. For microscopic examination, the specimens were dehydrated, cleaned, and mounted.

### Immunofluorescence

Tissue specimens were blocked in 5% bovine serum albumin for 30 min at room temperature before overnight incubation with the primary antibody at 4 °C. Fluorescence-conjugated secondary antibody was then incubated for 1 h at room temperature in the dark. The primary antibodies included CD11b antibody (Thermo Fisher Scientific Cat# 14–0112-82, RRID:AB_467108), Ly-6G/Ly-6C antibody (Thermo Fisher Scientific Cat# 14–5931-82, RRID:AB_467730), Cytokeratin 19 antibody (Proteintech Cat# 10,712–1-AP, RRID:AB_2133325). The nuclei were visualized using DAPI (C1006, Beyotime Biotechnology, Shanghai, China). The confocal microscope (LSM710, Zeiss, Jena, Germany) was used to obtain fluorescence images.

### Enzyme-linked immunosorbent assay (ELISA)

According to the manufacturer’s instructions, CXCL1 levels in mouse plasma or tumor tissue were measured using an enzyme-linked immunosorbent assay kit for chemokine (C-X-C Motif) ligand 1 (SEA041Mu, Cloud-Clone Corp. Katy, TX, USA). Triplicate independent experiments were repeated.

### Flow cytometry analysis

Single-cell suspensions were collected and isolated from the spleen, tumor, lung, and peripheral blood of mice. The erythrocytes in tissue samples were lysed with ammonium chloride (BD Biosciences, San Jose, CA, USA). For flow cytometry analysis, cells were labeled with the corresponding antibodies, including CD8-APC/Cyanine7 (BioLegend Cat# 100,714, RRID:AB_312753), Gr-1-Alexa Fluor 488 (Thermo Fisher Scientific Cat# 53–5931-80, RRID:AB_469917), CD11b-PE (BioLegend Cat# 101,208, RRID:AB_312791), F4/80-APC (Thermo Fisher Scientific Cat# 17–4801-82, RRID:AB_2784648), CD206-PE-Cyanine7 (Thermo Fisher Scientific Cat# 25–2069-42, RRID:AB_2573426), CXCR2-PE (Thermo Fisher Scientific Cat# 12–1829-42, RRID:AB_11041903). The expression levels of perforin and granzyme B in CD8^+^ T cells were analyzed using the Perforin-PE (Thermo Fisher Scientific Cat# 12–9392-82, RRID:AB_466243) and Granzyme B-APC (Thermo Fisher Scientific Cat# 17–8898-82, RRID:AB_2688068) antibodies. 4T1 cell apoptosis was detected by flow cytometric analysis using Annexin V-PE/7-AAD apoptosis kit (70-APCC104, Multi Sciences, Hangzhou, China) according to the manufacturer’s instructions. Flow cytometry data was acquired on a FACS Aria III flow cytometer (BD Biosciences, San Jose, CA, USA) and a NovoCyte Quanteon Flow cytometer (Agilent Technologies, Santa Clara, CA, USA). Triplicate independent experiments were repeated.

### Statistical analysis

Data were presented as mean ± standard deviation (SD). Student’s t-test analysis or one-way ANOVA was applied. The Dunnett test was used as a post hoc test for dose-dependent data, and the Bonferroni post hoc test was applied to others. For repeated measurement data, repeated measures analysis of variance was used. The nonparametric test was used if data were not normally distributed. *P* < 0.05 was considered statistically significant. Statistical analysis was performed using SPSS software (IBM SPSS Statistics, version 26.0).

## Results

### Chronic psychological stress promotes the formation of an immunosuppressive microenvironment in breast cancer

**Fig. 1:**
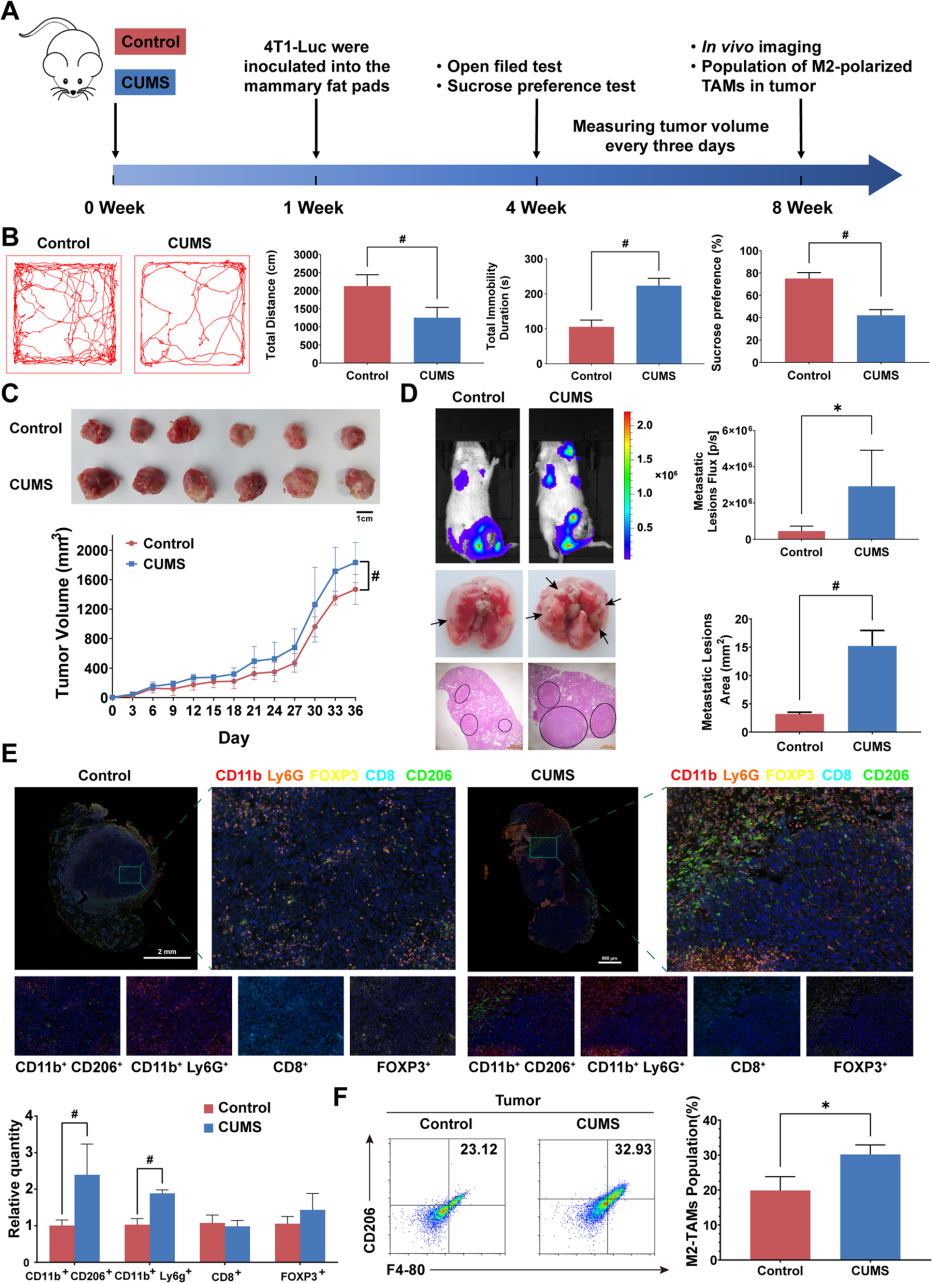
Chronic unpredictable mild stress (CUMS) promotes the formation of an immunosuppressive microenvironment in breast cancer. **A** Flowchart of the mouse experiments. **B** The representative mouse movement trajectories of each group in the open field tests were shown. Total immobility duration, total distance in the open field tests, as well as the percentage of sucrose water consumed in the sucrose preference tests were calculated (*n* = 6). **C** Representative pictures and growth curves of tumors in each group (*n* = 6). **D** In vivo imaging of lung metastatic lesions and the quantification of bioluminescence intensity in each group (*n* = 5, upper panel). Representative images of lung metastasis lesions in gross and HE staining (black circle indicates the metastatic lesions of the lung, and the scale bars indicate 1000 μm) and the quantification of metastatic lesion area in each group (*n* = 3, lower panel). **E** Representative multiplexed immunofluorescence images showing the tumor immune microenvironment of control (scale bar 2000 μm) and CUMS groups (scale bar 800 μm), using antibodies CD11b, Ly6G, FOXP3, CD8, and CD206. The proportion of CD11b^+^CD206^+^, CD11b^+^Ly6G^+^, CD8^+^, and FOXP3^+^ were quantified (*n* = 6). **F** Flow cytometry analysis of F4-80^+^ CD206^+^-TAMs population in the tumor tissue. Data are represented as the Mean ± SD. For statistical analysis, unpaired t-tests (**B**, **D**, **F**) and repeated measures analysis of variance (**C**) were applied. ^*^P < 0.05, ^#^*P* < 0.01

Chronic unpredictable mild stress (CUMS) is considered as a characterized rodent model that most closely mimics the behavioral and biochemical abnormalities induced by chronic psychological stress in the human social environment [34]. Here, we treated 4T1 tumor-bearing mice with CUMS to investigate the effect of chronic psychological stress on breast cancer progression. BABL/c mice were inoculated with 4T1 cells orthotopically in the mammary fat pads after 7 consecutive days of CUMS exposure, and the behavioral assays were performed on day 28. The breast cancer growth and metastasis were examined at the end of the experiment. The experimental flow chart is shown in Fig. 1A. Behavioral assays showed that chronic psychological stress decreased the total distance traveled by mice and increased immobility duration in the open field test. The daily sucrose consumption was also reduced (Fig. 1B). All these results indicate the successful establishment of the CUMS model. Moreover, chronic psychological stress promoted breast cancer growth (Fig. 1C), and the in vivo luciferase imaging assay indicated that lung metastasis was simultaneously aggravated (Fig. 1D). Consistently, the number and area of lung metastatic foci were also elevated in stressed mice (Fig. 1D). In order to evaluate the change of tumor microenvironment response to psychological stress, we conducted a multiplex immunofluorescence staining including CD11b, Ly6G, FOXP3, CD8, and CD206. It was found that the populations of CD11b^+^CD206^+^TAMs and CD11b^+^Ly6G^+^MDSCs were significantly increased following CUMS administration, suggesting that chronic psychological stress can aggravate the immunosuppressive TME in breast cancer (Fig. 1E). Moreover, flow cytometry further confirmed the upregulation of M2-type TAMs in the primary tumor (Fig. 1F). Therefore, CUMS may promote breast cancer progression via the elevation of TAMs.

### Chronic psychological stress promotes the expression and secretion of CXCL1 in TAMs

**Fig. 2:**
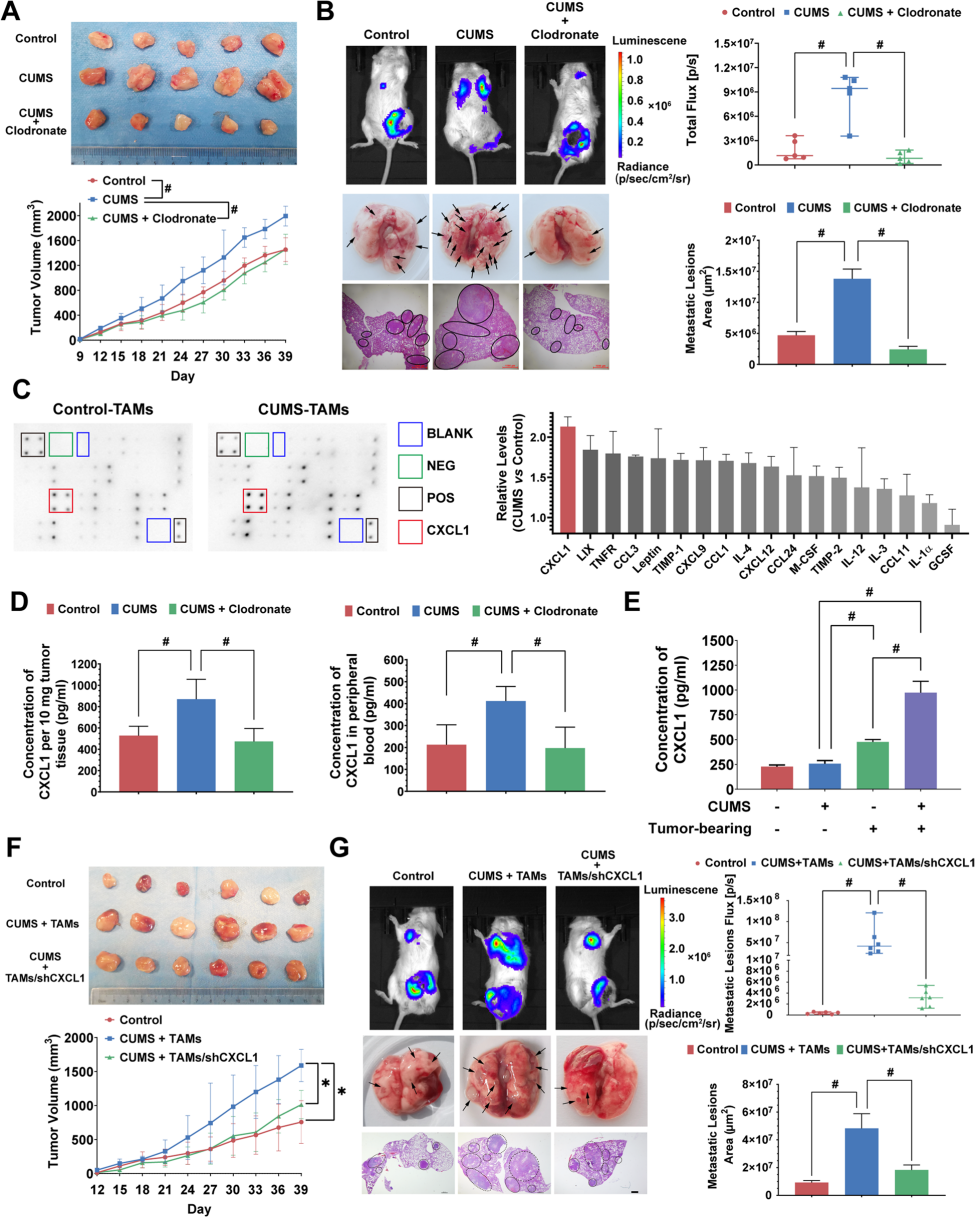
TAMs/CXCL1 signaling is crucial in mediating CUMS-induced breast cancer progression. **A** TAMs in tumor tissue were cleared by clodronate liposomes. Representative pictures and growth curves of tumors in each group were recorded (*n* = 5). **B** In vivo imaging of lung colonization and the quantification of bioluminescence intensity in each group (*n* = 5, upper panel). Representative images of lung metastasis lesions in gross and HE staining (black circle indicates the metastatic lesions of the lung, and the scale bars indicate 1000 μm) and the quantification of metastatic lesion area in each group (*n* = 3, lower panel). **C** TAMs were isolated from tumor tissues to detect chemokine release using cytokine microarray analysis. The chemokine levels for CUMS mice were measured relative to those of control mice. **D** CXCL1 levels in tumor tissues (*n* = 5) and peripheral blood (*n* = 8) were measured by ELISA. **E** Effects of tumor-bearing plus with or without CUMS exposure on CXCL1 levels in peripheral blood (*n* = 5). **F**-**G** 4T1 cells were co-injected with TAMs cells to establish breast cancer xenografts. The effects of CXCL1 knockdown (TAMs/shCXCL1) on tumor growth (*n* = 6), lung colonization (*n* = 6), and metastatic lesion area (*n* = 4) were investigated. Data are represented as the Mean ± SD. For statistical analysis, unpaired t-tests (**B**, **D**, **E**, **G**) and repeated measures analysis of variance (**A**, **F**) were applied. ^*^P < 0.05, ^#^*P* < 0.01

Because chronic psychological stress significantly increased the population of M2-TAMs, we used clodronate liposomes to ablate endogenous macrophages. Clodronate liposomes significantly inhibited breast cancer growth and lung metastasis induced by CUMS, resulting in the reduction of the number and area of lung metastatic foci (Fig. 2A–B). In addition, the cytokine microarray analysis indicated that CXCL1 was the highest upregulated chemokine in the TAM supernatants following CUMS treatment (Fig. 2C). It was also confirmed that CXCL1 levels in the tumor and peripheral blood were both significantly increased under CUMS, and clodronate liposomes administration could block CUMS-induced CXCL1 upregulation (Fig. 2D). Notably, CUMS did not increase CXCL1 levels in the non-tumor-bearing mice, suggesting that the elevated CXCL1 was primarily derived from breast cancer tissue (Fig. 2E). The following in vivo study further demonstrated that TAM co-injection with 4T1 cells significantly promoted breast cancer growth and metastasis, while CXCL1 silencing in TAMs remarkably inhibited the process (Fig. 2F–G). All these results indicate that TAM/CXCL1 signaling plays a crucial role in mediating CUMS-induced breast cancer progression.

### Chronic psychological stress regulates TAM/CXCL1 signaling through the GC/GR axis

**Fig. 3:**
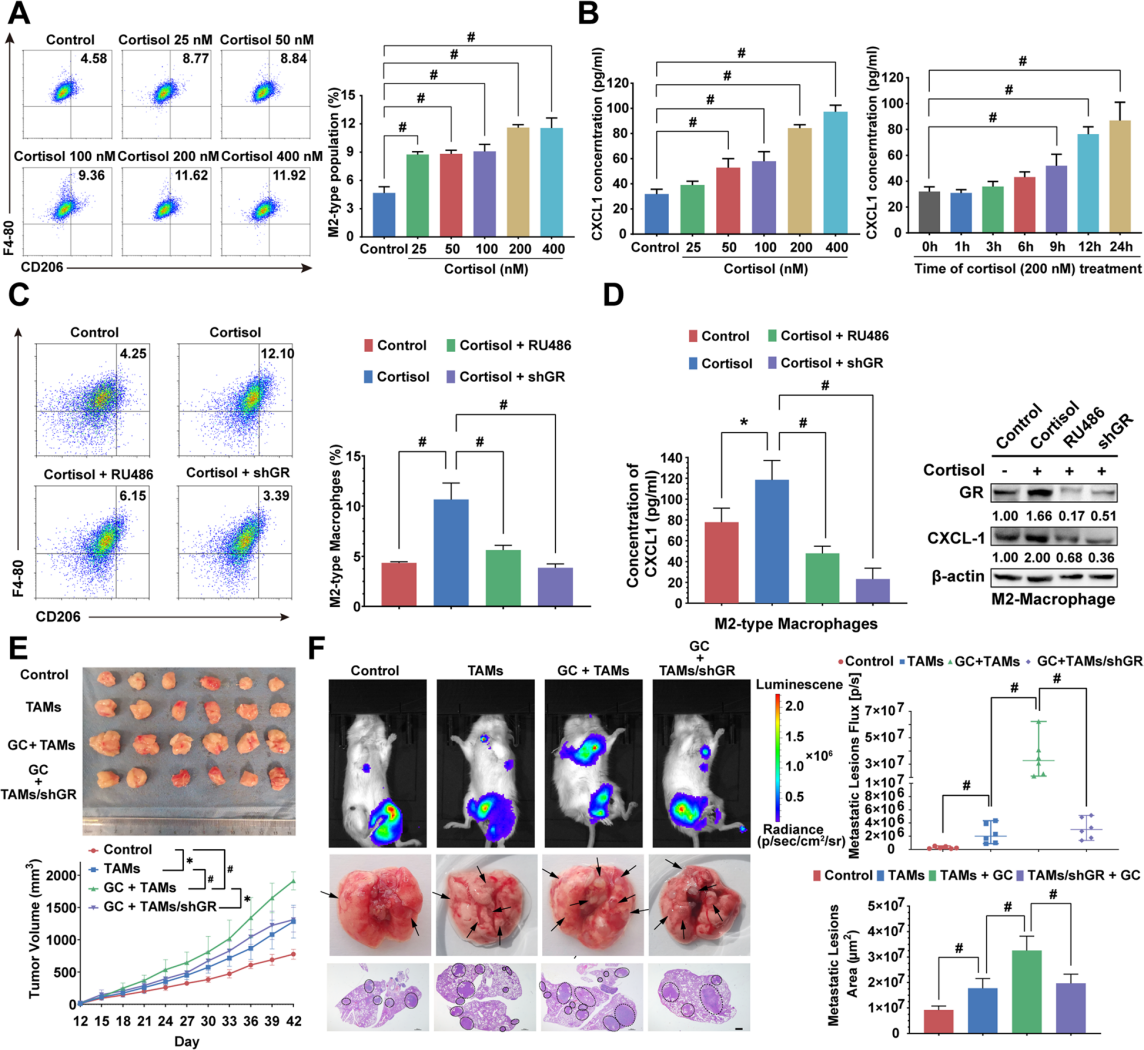
Cortisol triggers TAMs/CXCL1 signaling in a GR-dependent manner. **A** The population of CD206^+^-RAW264.7 after gradient cortisol treatment. **B** The CXCL1 level secreted by M2-polarized RAW264.7 was measured by ELISA assays after cortisol treatment. **C** The effects of GR antagonist RU486 or GR knockdown on cortisol-induced M2-polarization in RAW264.7 cells. **D** M2-polarized RAW264.7 cells were treated with GR antagonist RU486 or GR knockdown. The secretion and expression levels of CXCL1 induced by cortisol (200 nM) in M2-type RAW264.7 cells were then detected. **E**-**F** 4T1 cells were co-injected with TAMs or GR-knockdown TAMs into the mammary fat pad of mice to investigate the role of GR on cortisol-promoted cancer growth (*n* = 6), lung colonization (*n* = 6), and metastatic lesion formation (*n* = 4). Data are represented as the Mean ± SD. For statistical analysis, unpaired t-tests (**C**, **D**, **F**), one-way ANOVA with Dunnett post hoc test (**A**, **B**), and repeated measures analysis of variance (**E**) were applied. ^*^P < 0.05, ^#^*P* < 0.01

An elevated GC level is one of the landmark physiological events induced by chronic psychological stress. Flow cytometry analysis revealed that cortisol promoted M2-polarization of RAW264.7 cells in a dose-dependent manner (Fig. 3A). The enzyme-linked immuno sorbent assay also showed that cortisol significantly increased the CXCL1 level in a time- and dose-dependent manner (Fig. 3B). By contrast, the administration of GR antagonist RU486 or GR knockdown could inhibit the upregulation of M2 polarization and CXCL1 expression induced by cortisol treatment (Fig. 3C–D). The in vivo experiment further confirmed that GC administration could promote breast cancer growth and metastasis in 4T1-bearing mice. However, GR knockdown in TAMs significantly limited the stimulation effects of GC (Fig. 3E–F). All these results imply that CUMS-induced GC/GR activation is an upstream factor contributing to triggering TAM/CXCL1 signaling in the TME.

**Fig. 4:**
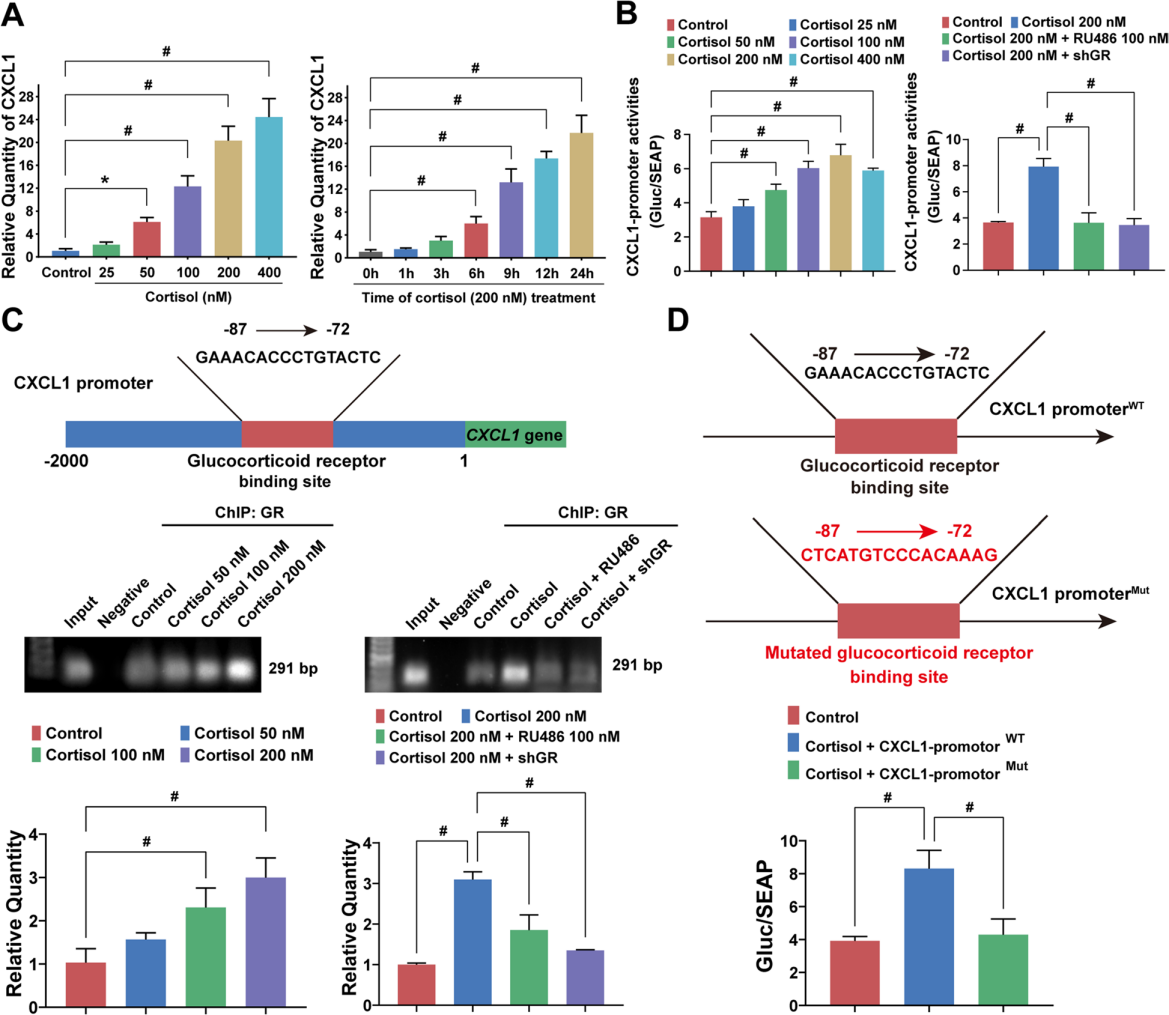
Cortisol enhances the interaction between GR with the CXCL1 promoter.** A** CXCL1 mRNA expression levels in M2-polarized RAW264.7 cells were measured by qPCR under cortisol treatment with various times and doses. **B** M2-polarized RAW264.7 cells were transfected with CXCL1 promoter-luciferase reporter plasmids that carried Gaussia luciferase (GLuc) and secreted alkaline phosphatase (SEAP) reporter gene. Gluc and SEAP activity of M2-polarized RAW264.7 cells treated with cortisol, GR antagonist (RU486) or shGR were measured using the luminometer. Results were displayed as ratios of GLuc and SEAP luminescence intensities. **C** Schematic diagram of the potential GR binding site on CXCL1 promoter predicted by hTFtarget database. The interaction between the CXCL1 promoter and GR was detected by Chromatin immunoprecipitation (ChIP) assay under cortisol, GR antagonist (RU486) or shGR treatment. **D** Schematic illustration of CXCL1 promoter mutation site (upper panel). Mutated CXCL1 promoter-luciferase reporter plasmids were introduced into the M2-polarized RAW264.7 cells. The luciferase activity was compared with wild-type CXCL1 promoter (lower panel). Data are represented as the Mean ± SD. For statistical analysis, unpaired t-tests (**B**, **C**, **D**), and one-way ANOVA with Dunnett post hoc test (**A**, **B**, **C**), were applied. ^*^P < 0.05, ^#^*P* < 0.01

We next investigated the underlying molecular mechanisms accounting for GR-mediated CXCL1 activation. A qPCR assay revealed that cortisol could enhance CXCL1 mRNA expression in a time- and dose-dependent manner (Fig. 4A). Moreover, the dual-luciferase reporter assay system also demonstrated that cortisol could activate the CXCL1 promoter directly, which was abrogated by GR antagonist RU486 or GR knockdown (Fig. 4B). The finding implies that GR plays a key role in activating CXCL1 transcription. Therefore, the potential GR binding site on the CXCL1 promoter was predicted using the hTFtarget database. Chromatin immunoprecipitation further verified that the − 87 to − 72 region of the CXCL1 promoter was a potential binding site for GR. Moreover, cortisol promoted the binding of GR to the CXCL1 promoter, while the GR antagonist or shRNA inhibited their interaction (Fig. 4C). By contrast, when the − 87 to − 72 region of the CXCL1 promoter was mutated, the cortisol-induced CXCL1 promoter activation was subsequently inhibited (Fig. 4D). Thus, CUMS-induced cortisol upregulation could activate CXCL1 transcription in TAMs by directly enhancing GR binding with the CXCL1 promoter.

### Chronic psychological stress promotes PMN formation

**Fig. 5:**
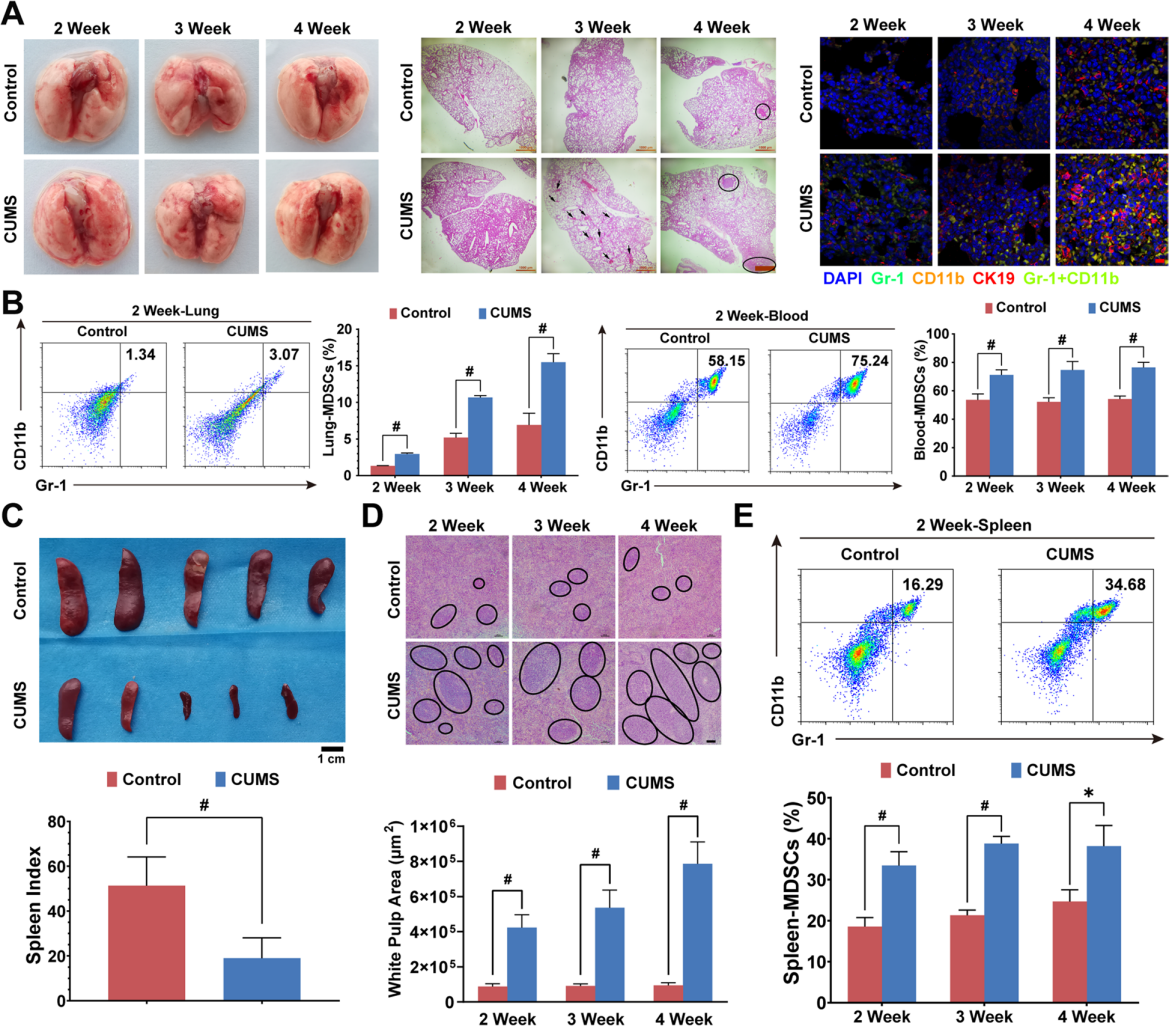
Chronic psychological stress promotes PMN formation.** A** Representative images of the lung in gross, HE staining (black circle indicates the metastatic lesions of the lung, and the scale bars indicate 1000 μm), and immunofluorescence (CK19 labeled cancer cells, Gr-1 and CD11b labeled MDSCs, the scale bars indicate 10 μm) for 2–4 weeks after orthotopic inoculation with 4T1 cells (*n* = 3). **B** MDSCs population in the lung and peripheral blood for 2–4 weeks after orthotopic inoculation were measured by flow cytometry analysis. **C** Pictures of the spleen in each group. The spleen index (spleen mass/body weight) was determined (*n* = 5). **D** Representative images of the white pulp in the spleen of each group (black circles). The white pulp areas were also calculated (*n* = 3). **E** MDSCs population in the spleen for 2–4 weeks after orthotopic inoculation was measured by flow cytometry analysis. Data are represented as Mean ± SD. For statistical analysis, unpaired t-tests were applied. ^*^P < 0.05, ^#^*P* < 0.01

PMN formation is considered a crucial step in establishing a metastatic lesion. Therefore, we continued to investigate the effects of chronic psychological stress on the formation of PMN. Little observable metastatic lesions were found in the lungs of mice during 2–4 weeks following orthotopic inoculation. However, the hematoxylin–eosin staining results show that chronic psychological stress could promote micro-metastatic lesion formation as early as the third week. More importantly, the immunofluorescence assay indicated that chronic psychological stress promoted the accumulation of CD11b^+^GR1^+^ MDSCs as early as the second week, validating that CUMS could promote PMN formation in breast cancer xenografts (Fig. 5A). MDSC is one of the representative immune-suppressive cells in the TME. The flow cytometry results further show that chronic psychological stress significantly increased the MDSC population in mouse lung tissue and peripheral blood. In particular, the MDSC population in the lung tissue was substantially increased over time (Fig. 5B). Notably, it was found that chronic psychological stress significantly reduced the spleen index and increased the white marrow area in the spleen, indicating the immune cell components in the spleen were significantly influenced by CUMS (Fig. 5C–D). Interestingly, chronic psychological stress also increased the MDSCs population in the spleen (Fig. 5E). As the spleen is considered as a vital immune regulation center responsible for MDSC recruitment and differentiation, it can be deduced that CUMS-mediated PMN formation may be associated with the spleen.

### Chronic psychological stress mobilizes spleen-derived MDSCs into the lung via CXCL1

**Fig. 6:**
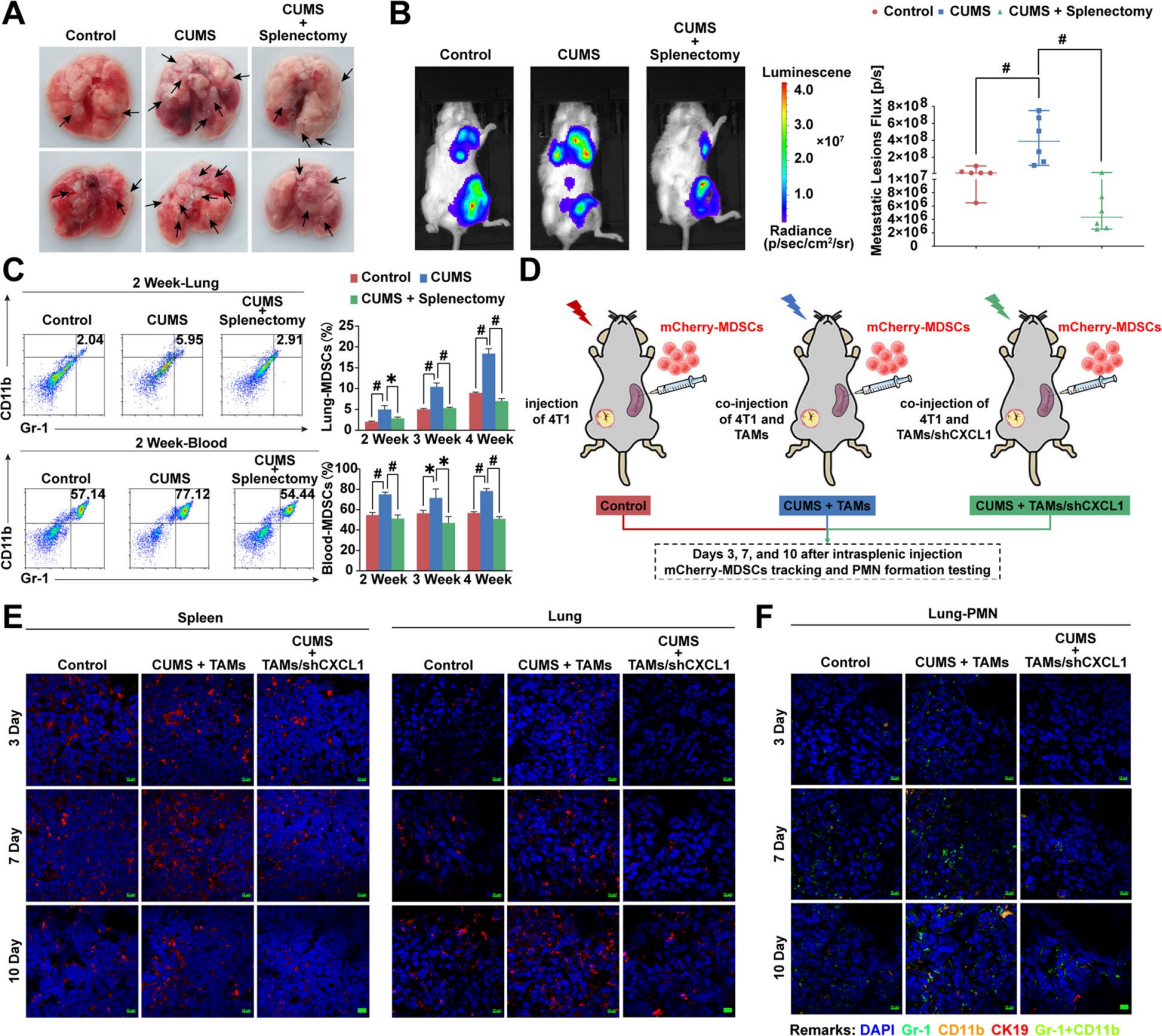
Chronic psychological stress mobilizes spleen-derived MDSCs into the lung via CXCL1.** A** Gross representative images of the lung metastatic lesions after splenectomy in CUMS-treated breast cancer xenografts (*n* = 3). **B** The fluorescence intensity of lung metastases after splenectomy in CUMS-treated breast cancer xenografts (*n* = 6). **C** MDSCs population in the lung and peripheral blood for 2–4 weeks after splenectomy in CUMS-treated breast cancer xenografts. **D** Flow diagram for tracing the distribution of splenic-derived MDSCs and PMN formation in mice. **E** The distribution of mCherry-labeled MDSCs in the spleen and lung was represented by immunofluorescence (the scale bars indicate 10 μm). **F** Representative immunofluorescence images for PMN formation in the lungs of mice injected with exogenous MDSCs via spleen (CK19 labeled cancer cells, Gr-1 and CD11b labeled MDSCs, the scale bars indicate 10 μm). Data are represented as Mean ± SD. For statistical analysis, unpaired t-tests were applied. ^*^P < 0.05, ^#^*P* < 0.01

In order to validate the key role of the spleen in mediating PMN formation induced by CUMS, we performed splenectomy on breast tumor-bearing mice. It was noticed that splenectomy significantly blocked CUMS-mediated lung metastasis, and the number of metastatic lesions and the fluorescence intensity of metastasis were both decreased (Fig. 6A–B). Moreover, the MDSC population in the lung and peripheral blood was also reduced in the splenectomy mice from the second to fourth weeks (Fig. 6C), suggesting that the spleen may be essential for MDSCs mobilized into the lung. Therefore, we sorted MDSCs from the spleen of CUMS-exposed mice bearing breast cancer, labeled them with mCherry, and subsequently injected them into the spleen of another breast-cancer-bearing mouse. The distribution of mCherry-MDSCs in the spleen, lung, and primary tumor was tracked on days 3, 7, and 10 after intrasplenic injection (Fig. 6D). The results show that CUMS gradually promoted the recruitment of exogenous mCherry-MDSCs into the lung. In addition, CUMS promoted the proliferation of mCherry-MDSCs in the spleen compared to the control group, but the number of mCherry-MDSCs was reduced over time, indicating that CUMS could promote MDSC migration. More importantly, CXCL1 knockdown in the primary tumor significantly attenuated the recruitment of exogenous mCherry-MDSCs into the lung, validating the crucial role of CXCL1 in mobilizing MDSCs (Fig. 6E). Correspondingly, CUMS-induced PMN formation was also relieved by CXCL1 knockdown (Fig. 6F). All these findings indicate that CXCL1-induced MDSC mobilization in the spleen acts as the key step in CUMS-mediated PMN formation.

### CXCL1/CXCR2 signaling regulates the proliferation, motility, and immunosuppressive activity of spleen-derived MDSCs

**Fig. 7:**
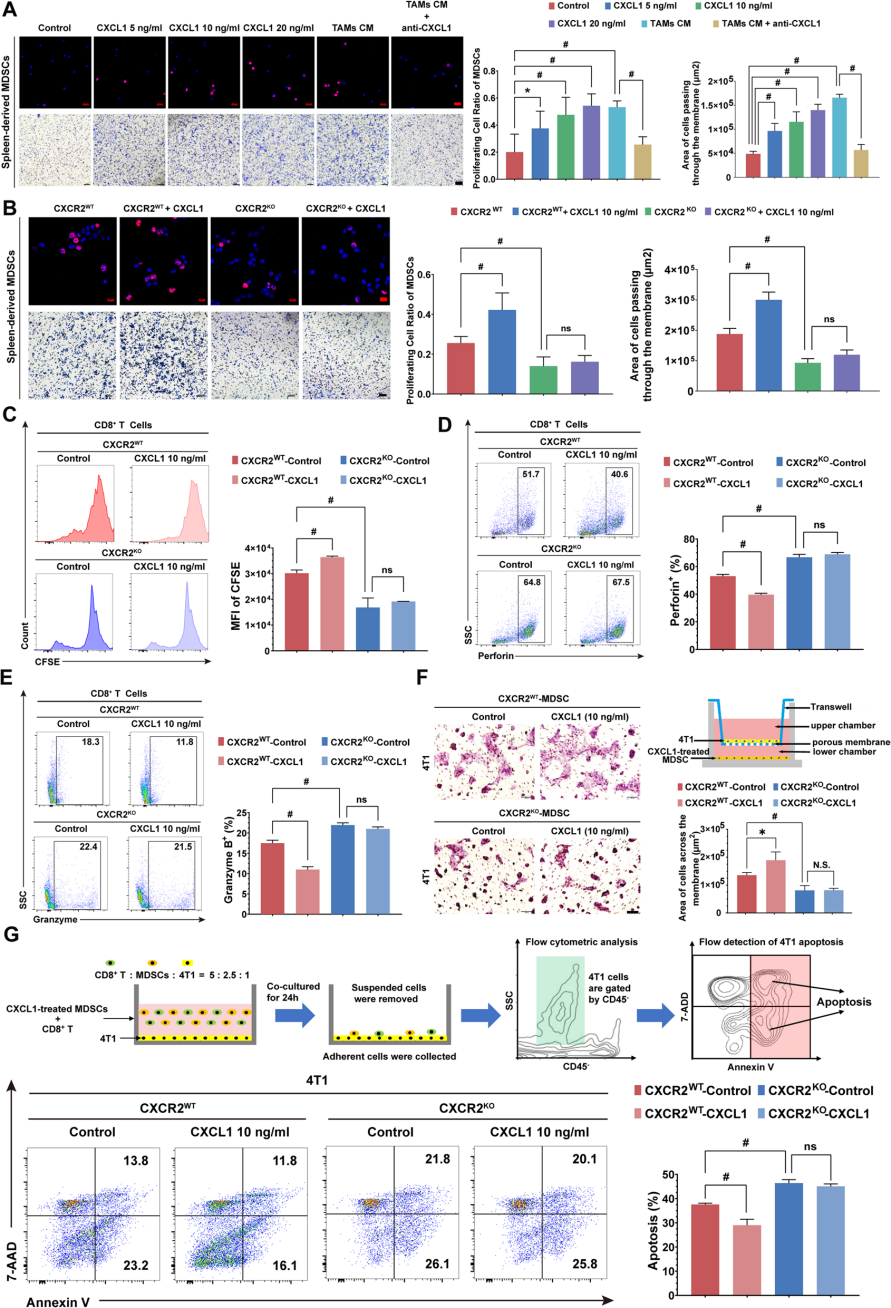
CXCL1/CXCR2 signaling regulates the proliferation, motility, and immunosuppressive activity of spleen-derived MDSCs.** A** MDSCs proliferation under different treatments for 24 h was determined by EdU assay. MDSCs motility was assessed using the Transwell chamber. **B** Proliferation and motility of spleen-derived MDSCs from CXCR2 knockout mice (CXCR2^KO^) or CXCR2 wild-type mice (CXCR2^WT^) were detected with or without CXCL1 (10 ng/ml) treatment for 24 h. **C** The proliferation of CD8^+^ T cells in the MDSCs (from CXCR2^WT^ or CXCR2^KO^ mice) co-culture system was detected by CFSE assay. **D**-**E** The granzyme B and perforin levels were measured in the MDSCs (from CXCR2^WT^ or CXCR2^KO^ mice) and CD8^+^ T cells co-culture system. **F** MDSCs were untreated or pretreated with CXCL1 (10 ng/ml) for 24 h. The capability of MDSCs to induce 4T1 migration was detected in the transwell co-culture system. **G** Flow diagram for 4T1 cell apoptosis detection (upper panel). MDSCs were untreated or pretreated with CXCL1 (10 ng/ml) for 24 h. MDSCs were then co-cultured with 4T1 and CD8^+^ T cells (CD8^+^T: MDSCs: 4T1 = 5: 2.5: 1) for 24 h. The 4T1 cells were collected and further distinguished by CD45^−^ gating. The ability of MDSCs inhibiting CD8^+^ T-induced apoptosis in 4T1 cells was measured using AnnexinV and 7-ADD labeling (lower panel). Data are represented as Mean ± SD. For statistical analysis, unpaired t-tests were applied. ^*^P < 0.05, ^#^*P* < 0.01

We next investigated the effects of CXCL1 on the biological activities of MDSCs. Spleen-derived MDSCs were treated with gradient concentrations of CXCL1 or the conditional culture medium (CM) from TAMs. Edu staining assay demonstrated that CXCL1 could dose-dependently activate the proliferation of MDSCs as well as the CM from TAMs. However, when the CXCL1 neutralizing antibody was administrated, the stimulation effects of TAMs-CM were abolished. A transwell assay also demonstrated that CXCL1 and TAM-CM could promote the motility of MDSCs (Fig. 7A). Notably, CXCL1-induced proliferation and motility of MDSCs were significantly diminished in CXCR2^KO^ mice compared with CXCR2^WT^ mice (Fig. 7B), suggesting that the stimulation effects of CXCL1 were CXCR2-dependent. Subsequently, we examined the effects of CXCL1/CXCR2 signaling on the immunosuppressive activity of MDSCs. CXCL1 enhanced the inhibition effects of MDSCs on CD8^+^ T cell proliferation, which was lost in MDSCs sorted from CXCR2^KO^ mice (Fig. 7C). In addition, the inhibition effects of MDSCs on perforin and granzyme B secretion from CD8^+^ T cells were enhanced by CXCL1 but diminished in CXCR2^KO^-MDSCs (Fig. 7D–E). In a transwell model of 4T1 cells and MDSCs, CXCL1-treated MDSCs were found to promote the invasion of 4T1 cells. However, the invasion-promoting effect was suppressed when CXCR2 was knocked out in MDSCs (Fig. 7F). Similarly, in the co-cultured system consisting of 4T1 cells, CXCR2^WT^-MDSCs, and CD8^+^T cells, CD8^+^T cell-induced apoptosis of 4T1 cells was reduced when MDSCs were treated with CXCL1 in advance. By contrast, the apoptosis induction effects of CD8^+^T cells were not affected when CXCR2 was knocked out in MDSCs (Fig. 7G), indicating that CXCL1 regulates the proliferation, motility, and immunosuppressive activity of MDSCs via CXCR2.

### Chronic psychological stress mobilizes spleen-derived MDSCs into the lung in a CXCR2-dependent manner

**Fig. 8:**
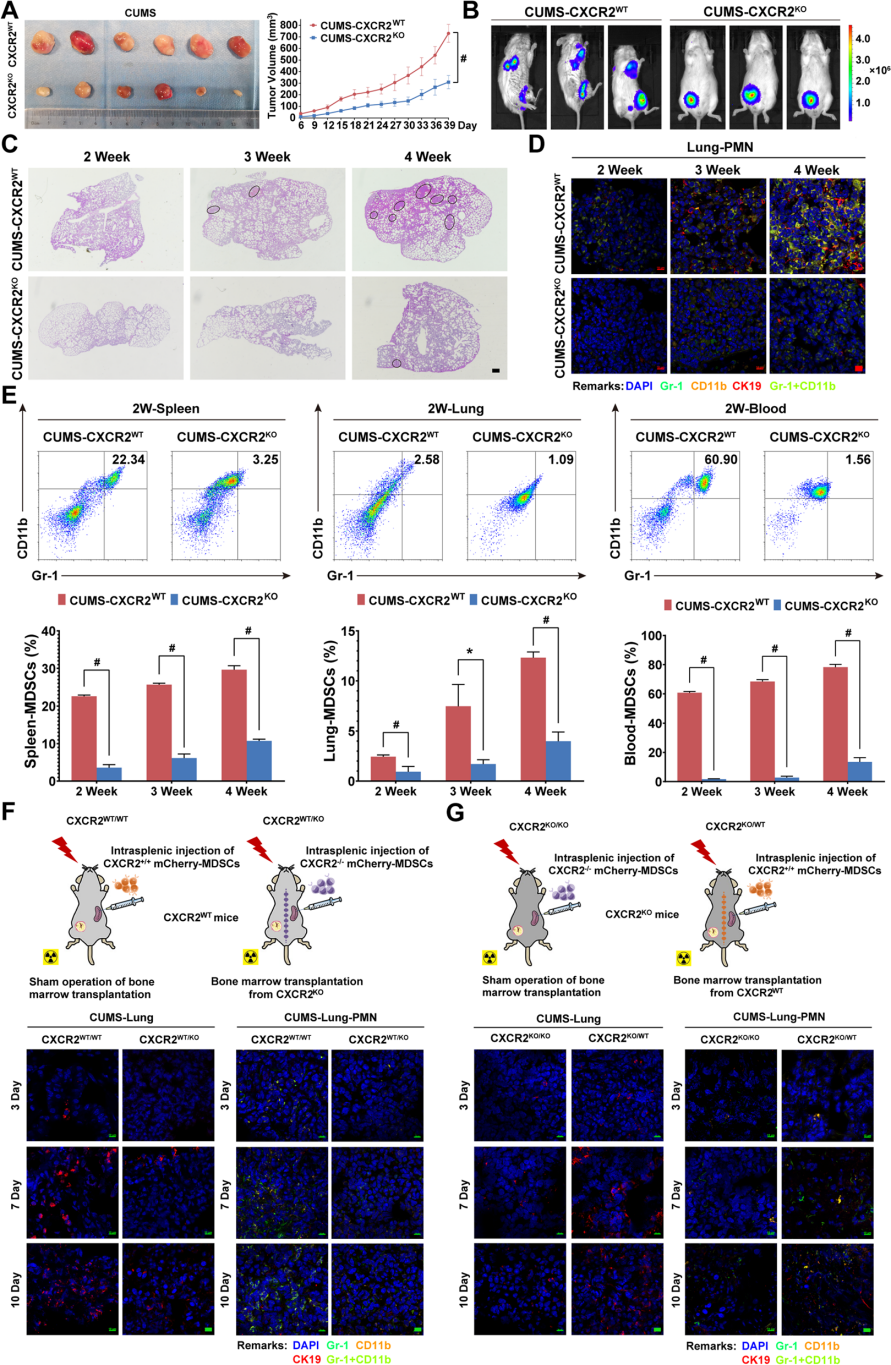
Chronic psychological stress mobilizes spleen-derived MDSCs into the lung in a CXCR2-dependent manner. **A** Representative pictures and growth curves of tumors in CXCR2^WT^ and CXCR2^KO^ mice under CUMS (*n* = 6). **B** The fluorescence intensity of lung metastases in CXCR2^WT^ and CXCR2^KO^ mice under CUMS (*n* = 3). **C** Representative lung images in HE staining for 2–4 weeks in CXCR2^WT^ and CXCR2^KO^ mice bearing breast cancer under CUMS (black circle indicates the metastatic lesions of the lung) (*n* = 3). **D** Representative immunofluorescence images for PMN formation for 2–4 weeks in the lungs of CXCR2^WT^ and CXCR2^KO^ mice bearing breast cancer under CUMS (CK19 labeled cancer cells, Gr-1 and CD11b labeled MDSCs, the scale bars indicate 10 μm). **E** MDSCs population in the lung, peripheral blood, and spleen of CXCR2^WT^ and CXCR2^KO^ mice bearing breast cancer under CUMS. **F** Flow diagram for establishing CXCR2^WT/KO^ bone marrow chimeric mice and follow-up detection. CXCR2^WT/WT^ mice that underwent sham surgical operation served as control (upper panel). Representative immunofluorescence images for the recruitment of exogenous mCherry-labeled MDSCs to the lung and PMN formation in the lung (lower panel). **G** Flow diagram for establishing CXCR2^KO/WT^ bone marrow chimeric mice and follow-up detection. CXCR2^KO/KO^ mice that underwent sham surgical operation served as control (upper panel). Representative immunofluorescence images for the recruitment of exogenous mCherry-labeled MDSCs to the lung and PMN formation in the lung (lower panel). Data are represented as Mean ± SD. For statistical analysis, repeated measures analysis of variance (**A**) and unpaired t-tests (**E**) were applied. ^*^P < 0.05, ^#^*P* < 0.01

Notably, CUMS-induced breast cancer growth and metastasis were remarkably attenuated in CXCR2^KO^ mice (Fig. 8A–C), and the accumulation of CD11b^+^GR1^+^-MDSCs in the lung was also simultaneously decreased (Fig. 8D). Furthermore, flow cytometry results show that the population of CD11b^+^GR1^+^-MDSCs in the spleen, lung, and peripheral blood of CXCR2^KO^ mice was significantly reduced (Fig. 8E), further confirming the crucial role of CXCL1/CXCR2 axis in regulating the bio-functions of MDSCs during breast cancer progression. In order to confirm the crucial role of CXCR2^+/+^-MDSCs in mediating PMN formation, we transplanted the bone marrow cells of CXCR2^−/−^ mice into CXCR2^+/+^ mice. 4T1 breast cancer cells were then orthotopically inoculated at the sixth week, followed by intrasplenic injection of mCherry-labeled CXCR2^+/+^ or CXCR2^−/−^ MDSCs. All mice were continuously exposed to CUMS. The distribution of mCherry-labeled MDSCs in the spleen and lung was tracked on 3, 7, and 10 days after intrasplenic injection. It was found that the recruitment of exogenous MDSCs to the lung was impaired in CXCR2^WT/KO^ bone marrow chimeric mice. Correspondingly, PMN formation was also relatively inhibited compared to CXCR2 ^WT/WT^ mice (Fig. 8F). By contrast, when the bone marrow cells were transplanted from CXCR2^+/+^ donor mice to CXCR2^−/−^ recipient mice, the MDSC recruitment and PMN formation was accelerated (Fig. 8G). These results validate the significance of CXCR2 in mobilizing splenic MDSCs during CUMS.

## Discussion

Immunity modulation has been considered a crucial hallmark in mediating chronic psychological stress-induced cancer initiation and development. In this study, we found that chronic psychological stress increased the population of TAMs, which secreted the chemokine CXCL1 and in turn facilitated the mobilization of splenic MDSCs into the lung PMN via CXCR2. Our previous study has found that CUMS could enhance breast cancer stemness by activating GRP78/LRP5/β-catenin signaling pathway, which was also mediated by increased cortisol [34]. However, the above phenomenon was just the direct effects of cortisol on breast cancer cells. It is more significant to study the influence of CUMS-induced cortisol on the cancer microenvironment. By utilizing multiplex immunofluorescence staining and chemokine array analysis, TAMs/CXCL1 was identified as one of the main immune-related signals responsive to CUMS or cortisol stimulation. Interestingly, TAMs/CXCL1 signal was also proved to enhance breast cancer stemness in the previous study [37, 38]. It is interesting to explore the interaction between CXCL1 and GRP78 in the future. Similarly, several studies also reported that stress-induced epinephrine elevation could polarize macrophages to the M2 phenotype via activating the adrenergic receptor β2 receptor [39]. The M2-like TAMs then facilitated cancer cell escape by secreting high levels of IL-10, VEGF, PGE2, and MMP-9. Moreover, the clearance of cancer cells by macrophages was also suppressed following psychological stress via disturbing the balance of the “eat me” signal receptor LRP1 and “don’t eat me” signal SIRPα on macrophages [40]. In particular, we found that stress-induced GCs could polarize macrophages into M2-like phenotype via GR signaling, indicating that GC/GR axis acted as the upstream switch responsible for macrophage infiltration and polarization. GC/GR signaling was demonstrated to suppress immunity in early research, and its role in tumor immune modulation was highlighted in recent findings [14, 41]. GC was found to promote TSC22D3 expression in dendritic cells, thereby blocking type-I IFN responses and IFNγ^+^ T-cell activation, which finally resulted in the compromise of therapy-induced anti-cancer immunosurveillance [42]. Notably, it was recently demonstrated that GR regulated PD-L1 and MHC-I in pancreatic cancer cells to promote immune escape and immunotherapy resistance [14]. In mouse models of pancreatic ductal adenocarcinoma, either tumor-cell-specific depletion or pharmacologic inhibition of GR led to PD-L1 downregulation and MHC-I upregulation, which in turn promoted the activity of cytotoxic T cells to enhance anti-tumor immunity. In pancreatic cancer patients, GR expression was also associated with high expression of PD-L1, low expression of MHC-I, and poor survival [14]. GR is among the nuclear receptor family members that bind with the promoter region to regulate gene transcription [43]. Our study demonstrated that GR was capable of binding with CXCL1 promoter and stimulating its transcription, finally resulting in CXCR2^+^ MDSC recruitment from the spleen. Interestingly, another study also reported that chronic psychological stress could induce higher expression of CXCR2 on myeloid cells via activating β-adrenergic receptor signaling in hepatocellular carcinoma. Moreover, it was also observed that MDSCs populations in the spleen were significantly elevated following chronic psychological stress, and β2 adrenergic receptor signaling was identified as the crucial mediator driving the immune suppressive activity of MDSCs [44]. Therefore, our study further established the bridge between the brain, tumor, and spleen via GC/GR-TAM/CXCL1-MDSC signaling.

It is generally accepted that the recruitment of MDSCs into the metastatic site is essential for PMN formation [45]. Recently, emerging studies have focused on how MDSCs are mobilized and recruited. Chemokines and chemokine receptors were shown to be crucial determinators. CCL12 was reported to attract MDSCs to the PMN in the lung of tumor-bearing mice [46]. The CCL2 blockade was also found to attenuate the mobilization of MDSCs into the lung PMN [47]. Moreover, CXCL1 could regulate the infiltration of MDSCs in the premetastatic liver tissue in colon cancer xenografts, while inhibition of its chemokine receptor CXCR2 suppressed the recruitment of MDSCs in the PMN [24]. Similarly, our study also found that chronic psychological stress-induced CXCL1 elevation led to MDSC recruitment into the lung tissue. CXCL1 derived from TAMs could promote the viability, migration, and immunosuppressive activities of MDSCs via CXCR2. However, chronic psychological stress-induced MDSC recruitment and PMN formation was significantly attenuated in CXCR2^KO^ mice. Some studies have suggested that CXCR2 is not only capable of regulating myeloid cells mobilization from bone marrow to peripheral circulation [48], but also of maintaining the survival/self-renewal of hematopoietic stem/progenitor cells [49]. CXCR2 inhibitors are now available in clinical trials as adjunctive cancer therapies. SX-682, for example, is an orally bioavailable CXCR2 inhibitor that blocks MDSC accumulation and promotes NK cell activation in head and neck cancer models. At present, clinical trials of SX-682 plus with anti-PD-1 (Pembrolizumab) in stage III and IV melanoma patients are ongoing (NCT03161431) [26, 50].

The spleen is not only an immunological and hematopoietic organ but also functions as a homeostatic regulator [51]. Chronic stress was previously reported to induce splenocyte apoptosis and immunosuppression via modulating the balance between Th1/Th2 cells. Consistently, our study also found that chronic stress resulted in a significant reduction of the spleen index, accompanied by the accumulation of MDSCs. Notably, splenectomy could reduce the frequency of MDSCs in the lung and peripheral blood that are elevated by chronic psychological stress. Interestingly, previous studies also found that splenectomy prolonged the survival time, inhibited tumor growth, and improved the immune status of tumor-bearing mice [52]. It is conceivable that hematopoiesis is increased to replenish the immune cell pool and restore homeostasis following splenectomy. Therefore, splenectomy may temporarily reduce the number of MDSCs and inhibit cancer growth [53]. Splenectomy was once considered a radical approach to reducing the number of MDSCs and eliminating their tumor-promoting functions [54]. However, the role of the spleen in tumor immunology is controversial and contradictory. Although splenectomy suppressed the tumor growth and metastasis of non-small cell lung cancer at the advanced stage, its anti-cancer effects were not observed at the early stage [55]. The phenomenon may be related to the cancer type, cancer stage, and animal xenografts. Therefore, the clinical significance of splenectomy in cancer patients is still to be explored and evaluated. Chemokines are considered a crucial factor in recruiting and modulating spleen-derived immunocytes. In the murine MCA203 fibrosarcoma model, CCL2 was identified as a crucial chemokine recruiting MDSCs in the spleen in a CCR2-dependent manner [56]. Similarly, we found that CXCL1 could promote the mobilization of splenic MDSCs into the lung in a CXCR2-dependent manner. Both CXCL1 silencing or CXCR2 knockout significantly impaired the process. Moreover, the population of MDSCs in the spleen was remarkably reduced in CXCR2^KO^ mice, indicating that CXCR2 plays a crucial role in modulating the immune function of splenocytes. A recent study also reported that chronic stress could enhance CXCR2 expression in the bone marrow MDSCs and promote them to mobilize into the spleen [44]. It is worth developing CXCR2 targeting strategies to modulate the spleen immunity to improve cancer prognosis in preclinical studies.

## Conclusion

Taken together, our study results not only demonstrate that chronic psychological stress promotes breast cancer progression via activating TAM/CXCL1 signaling in a GC/GR-dependent manner, but also highlight the role of CXCL1-CXCR2 signaling in mobilizing spleen MDSCs into the lung to establish PMN, thereby facilitating metastasis. Further research focusing on the role of spleen in mediating chronic psychological stress-induced breast cancer progression and developing targeting agents will be worthwhile.

## Data Availability

Data are available on reasonable request. All data generated or analyzed during this study are included either in this article or in the supplementary information files.

## Abbreviations

BMDCs: Bone-marrow-derived myeloid cells
CFSE: Carboxyfluorescein-succinimidyl-ester
ChIP: Chromatin immunoprecipitation
CM: Conditional culture medium
CXCL1: C–X–C motif ligand 1
CXCR2: CXC chemokine receptor 2
CUMS: Chronic unpredictable mild stress
EdU: 5-Ethynyl-2’-deoxyuridine
ELISA: Enzyme-linked immunosorbent assay
ER: Estrogen receptor
GCs: Glucocorticoids
GR: Glucocorticoid receptor
HRP: Horseradish peroxidase
MDSCs: Myeloid-derived suppressor cells
MSCs: Mesenchymal stromal cell
PMN: Pre-metastatic niche
PVDF: Polyvinylidene fluoride
SDS-PAGE: Sodium dodecyl sulfate polyacrylamide gel electrophoresis
TAMs: Tumor-associated macrophages
TDSFs: Tumor-derived secreted factors
TSA: Tyramine signal amplification
VEGF-A: Vascular endothelial growth factor A

## References

[1] Siegel RL, Miller KD, Fuchs HE, Jemal A (2022). Cancer statistics, 2022. CA Cancer J Clin.

[2] Waks AG, Winer EP (2019). Breast cancer treatment: a review. JAMA.

[3] Lu J, Steeg PS, Price JE, Krishnamurthy S, Mani SA, Reuben J (2009). Breast cancer metastasis: challenges and opportunities. Cancer Res.

[4] Kruk J, Aboul-Enein BH, Bernstein J, Gronostaj M (2019). Psychological stress and cellular aging in cancer: a meta-analysis. Oxid Med Cell Longev.

[5] Lillberg K, Verkasalo PK, Kaprio J, Teppo L, Helenius H, Koskenvuo M (2003). Stressful life events and risk of breast cancer in 10,808 women: a cohort study. Am J Epidemiol.

[6] Hanoun M, Maryanovich M, Arnal-Estape A, Frenette PS (2015). Neural regulation of hematopoiesis, inflammation, and cancer. Neuron.

[7] Wang X, Wang N, Zhong L, Wang S, Zheng Y, Yang B (2020). Prognostic value of depression and anxiety on breast cancer recurrence and mortality: a systematic review and meta-analysis of 282,203 patients. Mol Psychiatry.

[8] Sommershof A, Scheuermann L, Koerner J, Groettrup M (2017). Chronic stress suppresses anti-tumor TCD8+ responses and tumor regression following cancer immunotherapy in a mouse model of melanoma. Brain Behav Immun.

[9] Andersen BL, Farrar WB, Golden-Kreutz D, Kutz LA, MacCallum R, Courtney ME (1998). Stress and immune responses after surgical treatment for regional breast cancer. J Natl Cancer Inst.

[10] Mundy-Bosse BL, Thornton LM, Yang HC, Andersen BL, Carson WE (2011). Psychological stress is associated with altered levels of myeloid-derived suppressor cells in breast cancer patients. Cell Immunol.

[11] Eckerling A, Ricon-Becker I, Sorski L, Sandbank E, Ben-Eliyahu S (2021). Stress and cancer: mechanisms, significance and future directions. Nat Rev Cancer.

[12] Obradovic MMS, Hamelin B, Manevski N, Couto JP, Sethi A, Coissieux MM (2019). Glucocorticoids promote breast cancer metastasis. Nature.

[13] Landwehr LS, Altieri B, Schreiner J, Sbiera I, Weigand I, Kroiss M (2020). Interplay between glucocorticoids and tumor-infiltrating lymphocytes on the prognosis of adrenocortical carcinoma. J Immunother Cancer.

[14] Deng Y, Xia X, Zhao Y, Zhao Z, Martinez C, Yin W (2021). Glucocorticoid receptor regulates PD-L1 and MHC-I in pancreatic cancer cells to promote immune evasion and immunotherapy resistance. Nat Commun.

[15] Pan D, Kocherginsky M, Conzen SD (2011). Activation of the glucocorticoid receptor is associated with poor prognosis in estrogen receptor-negative breast cancer. Cancer Res.

[16] Coutinho AE, Chapman KE (2011). The anti-inflammatory and immunosuppressive effects of glucocorticoids, recent developments and mechanistic insights. Mol Cell Endocrinol.

[17] Gandhi S, Elkhanany A, Oshi M, Dai T, Opyrchal M, Mohammadpour H (2020). Contribution of immune cells to glucocorticoid receptor expression in breast cancer. Int J Mol Sci.

[18] Liu Y, Cao X (2016). Characteristics and significance of the pre-metastatic niche. Cancer Cell.

[19] Wang Y, Ding Y, Guo N, Wang S (2019). MDSCs: key criminals of tumor pre-metastatic niche formation. Front Immunol.

[20] Li R, Wen A, Lin J (2020). Pro-inflammatory cytokines in the formation of the pre-metastatic niche. Cancers (Basel).

[21] Sceneay J, Parker BS, Smyth MJ, Moller A (2013). Hypoxia-driven immunosuppression contributes to the pre-metastatic niche. Oncoimmunology.

[22] Zheng Y, Wang N, Wang S, Yang B, Situ H, Zhong L (2020). XIAOPI formula inhibits the pre-metastatic niche formation in breast cancer via suppressing TAMs/CXCL1 signaling. Cell Commun Signal.

[23] Yu PF, Huang Y, Han YY, Lin LY, Sun WH, Rabson AB (2017). TNFα-activated mesenchymal stromal cells promote breast cancer metastasis by recruiting CXCR2(+) neutrophils. Oncogene.

[24] Wang D, Sun H, Wei J, Cen B, DuBois RN (2017). CXCL1 is critical for premetastatic niche formation and metastasis in colorectal cancer. Cancer Res.

[25] Katoh H, Wang D, Daikoku T, Sun H, Dey SK, Dubois RN (2013). CXCR2-expressing myeloid-derived suppressor cells are essential to promote colitis-associated tumorigenesis. Cancer Cell.

[26] Yang J, Yan C, Vilgelm AE, Chen SC, Ayers GD, Johnson CA (2021). Targeted deletion of CXCR2 in myeloid cells alters the tumor immune environment to improve antitumor immunity. Cancer Immunol Res.

[27] Maenhout SK, Thielemans K, Aerts JL (2014). Location, location, location: functional and phenotypic heterogeneity between tumor-infiltrating and non-infiltrating myeloid-derived suppressor cells. Oncoimmunology.

[28] Cassetta L, Baekkevold ES, Brandau S, Bujko A, Cassatella MA, Dorhoi A (2019). Deciphering myeloid-derived suppressor cells: isolation and markers in humans, mice and non-human primates. Cancer Immunol Immunother.

[29] Corzo CA, Condamine T, Lu L, Cotter MJ, Youn JI, Cheng P (2010). HIF-1alpha regulates function and differentiation of myeloid-derived suppressor cells in the tumor microenvironment. J Exp Med.

[30] Youn JI, Nagaraj S, Collazo M, Gabrilovich DI (2008). Subsets of myeloid-derived suppressor cells in tumor-bearing mice. J Immunol.

[31] Tavukcuoglu E, Horzum U, Yanik H, Uner A, Yoyen-Ermis D, Nural SK (2020). Human splenic polymorphonuclear myeloid-derived suppressor cells (PMN-MDSC) are strategically located immune regulatory cells in cancer. Eur J Immunol.

[32] Elpek KG, Cremasco V, Shen H, Harvey CJ, Wucherpfennig KW, Goldstein DR (2014). The tumor microenvironment shapes lineage, transcriptional, and functional diversity of infiltrating myeloid cells. Cancer Immunol Res.

[33] De Wispelaere W, Annibali D, Tuyaerts S, Lambrechts D, Amant F (2021). Resistance to immune checkpoint blockade in uterine leiomyosarcoma: what can we learn from other cancer types?. Cancers (Basel).

[34] Zheng Y, Zhang J, Huang W, Zhong LLD, Wang N, Wang S (2021). Sini san inhibits chronic psychological stress-induced breast cancer stemness by suppressing cortisol-mediated GRP78 activation. Front Pharmacol.

[35] Hare BD, Shinohara R, Liu RJ, Pothula S, DiLeone RJ, Duman RS (2019). Optogenetic stimulation of medial prefrontal cortex Drd1 neurons produces rapid and long-lasting antidepressant effects. Nat Commun.

[36] Zhang J, Wang N, Zheng Y, Yang B, Wang S, Wang X, Pan B, Wang Z. Naringenin in Si-Ni-San formula inhibits chronic psychological stress-induced breast cancer growth and metastasis by modulating estrogen metabolism through FXR/EST pathway. J Adv Res. 2023;47:189-207. 10.1016/j.jare.2022.06.006 PMC10173160 35718080

[37] Ciummo SL, D'Antonio L, Sorrentino C, Fieni C, Lanuti P, Stassi G (2021). The C-X-C motif chemokine ligand 1 sustains breast cancer stem cell self-renewal and promotes tumor progression and immune escape programs. Front Cell Dev Biol.

[38] Wang S, Liu X, Huang R, Zheng Y, Wang N, Yang B (2019). XIAOPI formula inhibits breast cancer stem cells via suppressing tumor-associated macrophages/C-X-C motif chemokine ligand 1 pathway. Front Pharmacol.

[39] Qin JF, Jin FJ, Li N, Guan HT, Lan L, Ni H (2015). Adrenergic receptor β2 activation by stress promotes breast cancer progression through macrophages M2 polarization in tumor microenvironment. BMB Rep.

[40] Wu Y, Luo X, Zhou Q, Gong H, Gao H, Liu T (2022). The disbalance of LRP1 and SIRPα by psychological stress dampens the clearance of tumor cells by macrophages. Acta Pharm Sin B.

[41] Maeda N, Maruhashi T, Sugiura D, Shimizu K, Okazaki IM, Okazaki T (2019). Glucocorticoids potentiate the inhibitory capacity of programmed cell death 1 by up-regulating its expression on T cells. J Biol Chem.

[42] Yang H, Xia L, Chen J, Zhang S, Martin V, Li Q (2019). Stress-glucocorticoid-TSC22D3 axis compromises therapy-induced antitumor immunity. Nat Med.

[43] Cain DW, Cidlowski JA (2017). Immune regulation by glucocorticoids. Nat Rev Immunol.

[44] Cao M, Huang W, Chen Y, Li G, Liu N, Wu Y (2021). Chronic restraint stress promotes the mobilization and recruitment of myeloid-derived suppressor cells through β-adrenergic-activated CXCL5-CXCR2-Erk signaling cascades. Int J Cancer.

[45] Safarzadeh E, Orangi M, Mohammadi H, Babaie F, Baradaran B (2018). Myeloid-derived suppressor cells: Important contributors to tumor progression and metastasis. J Cell Physiol.

[46] Shi H, Zhang J, Han X, Li H, Xie M, Sun Y (2017). Recruited monocytic myeloid-derived suppressor cells promote the arrest of tumor cells in the premetastatic niche through an IL-1β-mediated increase in E-selectin expression. Int J Cancer.

[47] Eisenblaetter M, Flores-Borja F, Lee JJ, Wefers C, Smith H, Hueting R (2017). Visualization of tumor-immune interaction - target-specific imaging of S100A8/A9 reveals pre-metastatic niche establishment. Theranostics.

[48] Eash KJ, Greenbaum AM, Gopalan PK, Link DC (2010). CXCR2 and CXCR4 antagonistically regulate neutrophil trafficking from murine bone marrow. J Clin Invest.

[49] Sinclair A, Park L, Shah M, Drotar M, Calaminus S, Hopcroft LE (2016). CXCR2 and CXCL4 regulate survival and self-renewal of hematopoietic stem/progenitor cells. Blood.

[50] Tang F, Tie Y, Hong W, Wei Y, Tu C, Wei X (2021). Targeting myeloid-derived suppressor cells for premetastatic niche disruption after tumor resection. Ann Surg Oncol.

[51] Mebius RE, Kraal G (2005). Structure and function of the spleen. Nat Rev Immunol.

[52] Li B, Zhang S, Huang N, Chen H, Wang P, Li J (2016). Dynamics of the spleen and its significance in a murine H22 orthotopic hepatoma model. Exp Biol Med (Maywood).

[53] Mastio J, Condamine T, Dominguez G, Kossenkov AV, Donthireddy L, Veglia F (2019). Identification of monocyte-like precursors of granulocytes in cancer as a mechanism for accumulation of PMN-MDSCs. J Exp Med.

[54] Levy L, Mishalian I, Bayuch R, Zolotarov L, Michaeli J, Fridlender ZG (2015). Splenectomy inhibits non-small cell lung cancer growth by modulating anti-tumor adaptive and innate immune response. Oncoimmunology.

[55] Sevmis M, Yoyen-Ermis D, Aydin C, Bilgic E, Korkusuz P, Uner A (2017). Splenectomy-induced leukocytosis promotes intratumoral accumulation of myeloid-derived suppressor cells, angiogenesis and metastasis. Immunol Invest.

[56] Ugel S, Peranzoni E, Desantis G, Chioda M, Walter S, Weinschenk T (2012). Immune tolerance to tumor antigens occurs in a specialized environment of the spleen. Cell Rep.

